# Antimicrobial Susceptibility and Molecular Mechanisms of Fosfomycin Resistance in Clinical *Escherichia coli* Isolates in Mainland China

**DOI:** 10.1371/journal.pone.0135269

**Published:** 2015-08-07

**Authors:** Ya Li, Bo Zheng, Yun Li, Sainan Zhu, Feng Xue, Jian Liu

**Affiliations:** 1 Institute of Clinical Pharmacology, Peking University First Hospital, Beijing, PR of China; 2 Department of Biostatistics, Peking University First Hospital, Beijing, PR of China; University of Malaya, MALAYSIA

## Abstract

*Escherichia coli* is one of the most common pathogens in nosocomial and community-acquired infections in humans. Fosfomycin is a broad-spectrum antibiotic which inhibits peptidoglycan synthesis responsible for bacterial cell wall formation. Although low, the exact *E*. *coli* susceptibility to fosfomycin as well as the mechanisms of resistance in the population from Mainland China are mostly unknown. 1109 non-duplicate clinical *E*. *coli* strains isolated from urine, sputum, blood and pus samples in 20 widely dispersed tertiary hospitals from Mainland China were collected from July 2009 to June 2010, followed by determination of minimum inhibitory concentrations of fosfomycin. Detection of the *murA*, *glpT*, *uhpT*, *fosA*, *fosA*_*3*_ and *fosC* genes was performed in fosfomycin non-susceptible *E*. *coli* strains and conjugation experiments were employed to determine the mobility of *fosA*_*3*_ gene. In this study, 7.8% (86/1109) *E*. *coli* strains were fosfomycin non-susceptible. Amino acid substitutions in GlpT and MurA were found in six and four *E*.*coli* strains, respectively, while the *uhpT* gene was absent in eighteen *E*.*coli* strains. Twenty-nine isolates carried the transferable plasmid with the *fosA*_*3*_ gene at high frequencies of around 10^−6^ to 10^−7^ per donor cell in broth mating. The majority of isolates were susceptible to fosfomycin, showing that the drug is still viable in clinical applications. Also, the main mechanism of *E*. *coli* resistance in Mainland China was found to be due to the presence of the *fosA*_*3*_ gene.

## Introduction

*Escherichia coli* is one of the most common pathogens in nosocomial and community-acquired infections in humans. Fosfomycin is a broad-spectrum antibacterial agent against Gram-positive and Gram-negative bacteria; it inhibits the UDP-N-acetylglucosamine enolpyruvyl transferase (MurA), responsible for the formation of UDP-GlcNAc-enolpyruvate in the biosynthesis of cell-wall peptidoglycans [1,2]. Fosfomycin enters the *E*. *coli* cell by using one of two transport systems: the glycerol-3-phosphate transport system (GlpT) or the hexose phosphate transport system (UlpT) [3,4]. The prevalence of fosfomycin resistance in *E*. *coli* remains low [5–8], but several resistance mechanisms to fosfomycin have been reported. Mutations in the *glpT* or *uhpT* genes can decrease the uptake of fosfomycin via the transport systems and give rise to fosfomycin resistance [4,9,10]. Resistance against fosfomycin can also be conferred by mutations in the *murA* gene, resulting in either reduced affinity between MurA and fosfomycin or through the over-expression of MurA [11,12]. In addition, the presence of the plasmid-borne *fosA*, *fosA*_*3*_ and *fosC*_*2*_ genes, which encode fosfomycin-modifying enzymes, can inactivate the drug by catalyzing the covalent addition of glutathione to fosfomycin [13].

However, little is known about the rate of resistance or *E*. *coli* mechanisms of resistance to fosfomycin in Mainland China. Thus, here we investigated several fosfomycin resistance mutations and the mobility of the *fosA3* gene.

## Material and Methods

### Bacterial strains

A total of 1109 non-duplicate *E*. *coli* strains isolated from urine, sputum, blood and pus samples were collected as part of standard patient care from July 2009 to June 2010 in 20 widely dispersed tertiary hospitals. *E*. *coli* ATCC 25922 [14] was used for quality control for susceptibility testing and the sodium azide-resistant *E*. *coli* J53 [15] strain was used as a recipient in the mating experiment.

### Ethics

The samples were collected from hospitals participating in the Ministry of Health National Antimicrobial Resistance Surveillance Net (Mohnarin) study. No ethical approval or informed consent was required due to the retrospective nature of the study. All patient identifiable information was removed from the samples before the authors received them for analysis. The authors were not involved in the treatment and had no direct contact with the patients.

### Susceptibility testing

Susceptibility to antimicrobial agents was determined using the agar dilution method according to the Clinical and Laboratory Standards Institute [14]. The following antimicrobial agents were tested: fosfomycin trometamol, piperacillin-tazobactam, cefuroxime, cefotaxime, cefepime, imipenem, amikacin, levofloxacin, and nitrofurantoin. Based on minimum inhibitory concentrations (MIC), *E*. *coli* were classified as fosfomycin-susceptible (MIC ≤64mg/L), fosfomycin-intermediate (MIC = 128mg/L) and fosfomycin-resistant (MIC ≥256 mg/L). Extended-spectrum beta-lactamase (ESBL) producing isolates were detected according to previously described methods [14]. The reference strain *E*. *coli* ATCC 25922 was used as the positive control. The spontaneous fosfomycin resistance rate for *E*. *coli* J53 strain determined in our research was <10^−8^.

### PCR amplification of genes and sequence analysis

Detection of the *murA*, *glpT*, *uhpT*, *fosA*, *fosA*_*3*_ and *fosC* genes was performed in fosfomycin non-susceptible *E*. *coli* strains (intermediate and resistant *E*. *coli* strains; MIC ≥128 mg/L), and the primers used are listed in Table 1 [16–18]. Sequence analysis was performed with a Dye primer and a Dye Terminator cycle sequencing kit (Applied Biosystems) and with a 310 gene analyzer (ABI Prism).

**Table 1:** Oligonucleotide primers employed.

Amplified gene	Primer	Sequence	Amplicon size (bp)	Ref
*MurA*	MF	5'-AAACAGCAGACGGTCTATGG-3'	1260	19
	MR	5''-CCATGAGTTTATCGACAGAACG-3'		
*uhpT*	UF	5'-TTTTTGAACGCCCAGACACC-3'	1392	19
	UR	5'-AGTCAGGGGCTATTTGATGG-3'		
*glpT*	GF	5'-GCGAGTCGCGAGTTTTCATTG-3'	1359	19
	GR	5'-GGCAAATATCCACTGGCACC-3'		
*fosA*	FAF	5'-ATCTGTGGGTCTGCCTGTCGT-3'	271	16
	FAR	5'-ATGCCCGCATAGGGCTTCT-3'		
*fosC*_*2*_	FCF	5'-TGGAGGCTACTTGGATTTG-3'	217	16
	FCR	5'-AGGCTACCGCTATGGATTT-3'		
*fosA*_*3*_	FA3F	5'-GCGTCAAGCCTGGCATTT-3'	282	16
	FA3R	5'-GCCGTCAGGGTCGAGAAA-3'		

### Conjugation experiments

The conjugation experiments were carried out to determine the mobility of *fosA*_*3*_ gene from azide-sensitive isolates as donors to azide-resistant *E*. *coli* J53 as the recipient. Overnight cultures of 0.05 ml of the donor and 0.45 mL of the recipient strains were added to 3 mL of fresh Mueller Hinton (MH) broth (Oxoid Ltd., Basingstoke, Hampshire, United Kingdom), then incubated and gently stirred at 37°C for 12 hours. 0.1 mL of the mixtures were plated on MH agar (Oxoid Ltd., Basingstoke, Hampshire, United Kingdom) containing glucose-6-phosphate (25 mg/L), sodium azide (100 mg/L) and fosfomycin (Zambon Group, Milan, Italy) (32 mg/L) and incubated for 48 hours for selection. The conjugative transfer frequency was calculated as the ratio of the number of conjugants to the number of donors.

### Statistical analyses

Statistical tests were performed using Social Sciences software for Windows Version 14.0 (SPSS, Inc., Chicago, IL, USA). Enumeration data were expressed as percentage values. The differences in susceptibility between the groups were compared using the Chi-square test or Fisher’s exact test. The differences between the groups were considered significant if the p-values were smaller than 0.05 (two-sided test). The Bonferroni method was used to adjust the significant levels (0.05/3 = 0.0167) in multiple comparisons between any two levels of the susceptibility outcome.

## Results

1023/1109 (92.2%) of the *E*. *coli* isolates were susceptible to fosfomycin. The susceptibility rates of the isolates from urine, sputum, blood and pus samples were 95.0%, 87.1%, 93.3% and 93.3%, respectively. MIC_50_ of the strains from different specimen types were all 0.25 mg/L, whilst the MIC_90_ of the strains from urine, sputum, blood and pus samples were 4, 128, 16 and 32 mg/L, respectively (Table 2). The ESBL-positive rates were 67.2% (687/1023), 85.7% (30/35) and 90.2% (46/51) among fosfomycin-susceptible, intermediate and resistant isolates, respectively.

**Table 2:** Fosfomycin susceptibility of *E*. *coli* strains from different samples by source.

Sample source	No. of isolates	ESBL(%)	No. of isolates inhibited at fosfomycin MIC (mg/L) of														MIC_50_(mg/L)	MIC_90_(mg/L)	S (%)	I (%)	R (%)
			0.062	0.125	0.25	0.5	1	2	4	8	16	32	64	128	256	>256					
urine	262	65.9	7	59	116	34	15	4	4	1	4	4	1	3	8	2	0.25	4	95.0	1.2	3.8
sputum	264	77.9	7	47	94	27	23	9	10	5	2	2	4	15	12	7	0.25	128	87.1	5.7	7.2
blood	343	67.9	6	72	135	59	14	9	9	5	6	2	3	7	9	7	0.25	16	93.3	2.0	4.7
pus	240	63.3	5	46	90	38	19	5	4	3	3	6	5	10	3	3	0.25	32	93.3	4.2	2.5
Total	1109	68.8	25	224	435	158	71	27	27	14	15	14	13	35	32	19	0.25	32	92.2	3.2	4.6

ESBL—extended spectrum β-lactamases; MIC—minimum inhibitory concentration; MIC_50_ –minimum concentration that inhibits 50% of the growth; MIC_90_ –minimum concentration that inhibits 90% of the growth; S—susceptibility; I—intermediate; R—resistance

The antimicrobial resistance rates stratified by fosfomycin susceptibility categories are summarized in Table 3. Fosfomycin-resistant isolates were significantly more likely to be ESBL positive than were fosfomycin-susceptible isolates (P <0.001). In addition, the resistance rates for piperacillin-tazobactam, cefuroxime, cefotaxime, amikacin and nitrofurantoin were significantly higher in fosfomycin-resistant isolates than fosfomycin-susceptible isolates (P < 0.0167 for all comparisons).

**Table 3:** Antimicrobial resistance of *Escherichia coli* stratified by fosfomycin susceptibility.

		% resistant			*P* ^b^
		Fos-S (n = 1023)	Fos-I (n = 35)	Fos-R (n = 51)	
Agent^a^					
	TZP	3.8	11.4	13.7**	0.002
	CXM	71.1	94.3*	98.0**	< 0.001
	CTX	70.0	94.3*	98.0**	< 0.001
	FEP	34.1	48.6	49.0	0.023
	IMP	0.2	0	2.0	0.215
	AMK	4.2	37.1*	35.3**	< 0.001
	LVX	58.7	68.6	60.8	0.494
	TET	77.4	77.1	90.2	0.098
	NIT	1.4	14.3*	11.8**	< 0.001
ESBL positive		67.2	85.7	90.2**	< 0.001

Fos-S, fosfomycin—susceptible (MIC ≤64 mg/L); Fos-I, fosfomycin—intermediate (MIC = 128 mg/L); Fos-R, fosfomycin-resistant (MIC ≥256 mg/L); ESBL—extended spectrum β-lactamases.

^a^Drug abbreviations: FOS, fosfomycin trometamol; TZP, piperacillin-tazobactam;

CXM, cefuroxime; CTX, cefotaxime; FEP, cefepime; IMP, imipenem; AMK, amikacin; LVX, levofloxacin; TET, tetracycline; NIT, nitrofurantoin.

^b^ *P* value for comparison of resistance rates between fosfomycin-susceptible and fosfomycin-resistant isolates.

*compared to FOS-S, *P* < 0.0167

**compared to FOS-R, *P* < 0.0167

In 86 fosfomycin non-susceptible *E*. *coli* strains, amino-acid substitutions Val389Ile, Asp390Ala, Gln59Lys and Glu139Lys were found in MurA of *E*. *coli* strains J621, E261, E374 and R162, respectively. In addition, amino-acid substitutions Ile4Val and Gly84Asp were found in GlpT of *E*. *coli* strains H039 and R538, respectively. In strain Q446, the GlpT protein was truncated due to deletion of nucleotide 401A. Amino acid substitutions Thr144Pro and Pro173Ser were found in the GlpT of strains R046 and P305. Amino acid substitutions Ala12Val and Gln437Cys were found in GlpT of strains T229 and T297. The *uhpT* gene was absent from 18 of the test strains. 69 *E*. *coli* isolates were positive for *fosA*_*3*_, yet no *fosC*_*2*_ or *fosA* genes were detected among these isolates (Table 4). The *fosA*_*3*_ gene was able to transfer at frequencies varying from 1.1×10^−7^ to 9.9×10^−6^ between the donor and recipient in 29 isolates tested.

**Table 4:** Characterization of fosfomycin non-susceptible *E*. *coli* isolates.

*E*. *coli* strain	No. of isolates	MIC range (mg/L)	Amino acid substitutions or sequence variations^a^			*fosA*_*3*_
			GlpT	MurA	UhpT	
J443,J648,O241,L026,L088,Q097,D353,D503,G001,H256,A083,D360,R214,R494	14	128->256	None^c^	None	No peptide^b^	+
H039	1	256	Ile4Val	None	No peptide	+
K077,K105,N182,O013,O065,O250,P273L196,M094,M098,G198,R419,E042,E110,E169,E380,D224,D271,D440,D468,D542,A019,B050,C024,C049,C254,C282,Q079,Q108,R079,R057,Q008,Q056,F040,F070,G160,G383,D014,D265,T335,T436,T038,T108,T211,R259,R421,R143,R145	48	128->256	None	None	None	+
E261	1	128	None	Asp390Ala	None	+
E374	1	256	None	Gln59Lys	None	+
T229,T297	2	>256	Gly437Cys	None	None	+
R538	1	>256	Gly84Asp	None	None	+
R046	1	256	Thr144Pro,Pro173Ser	None	None	+
J609,L229,Q493,P303,Q091,G199,R120,R144,R206	9	128–256	None	None	None	−
Q446	1	128	Truncated to 206 aa (deletion of 401A)	None	None	−
P305	1	128	Thr144Pro,Pro173Ser 173Ser	None	None	−
R162	1	128	None	GlU139Lys	None	−
G182,G316	2	256	None	None	No peptide	−
J621	1	128	None	Val389Ile	No peptide	−

MIC—minimum inhibitory concentration; GlpT—glycerol-3-phosphate transport system; MurA—UDP-N-acetylglucosamine enolpyruvyl transferase; UlpT—hexose phosphate transport system.

^a^Genetic mutations are shown in brackets.

^b^Loss of the entire gene.

^c^No amino acid substitutions were found.

## Discussion

Fosfomycin has been introduced in clinical practice for about 30 years. However, this antibacterial agent is still not commonly used in China, and the fosfomycin resistance rate in clinical *E*. *coli* isolates remains very low [18]. In this study, only 7.8% (86/1109) of *E*. *coli* isolates were fosfomycin non-susceptible with a high ESBL-positive rate and levofloxacin-resistant rate. Among the fosfomycin-resistant isolates, the ESBL-positive rates were higher than in fosfomycin-susceptible isolates.

Fosfomycin inactivates the MurA enzyme by binding to the active site of its Cys-115 residue [19,20]. The amino acid Asp369Asn and Leu370Ile substitutions have been reported in fosfomycin- resistant *E*. *coli* isolates MSC17327 and MSC17323. Inspection of the crystal structure of *E*. *coli* MurA complexed with fosfomycin does not suggest an obvious role for Asp-369 and Leu-370 in the protein-inhibitor interaction [16]. In our study, the MurA substitutions of Val389Ile, Asp390Ala, Gln59Lys and Glu139Lys were found in four *E*. *coli* strains. However, further investigations are needed to find out whether the substitutions contribute to fosfomycin resistance.

Fosfomycin is transported into cells via two pathways: the glycerol-3-phosphate or the hexose phosphate transport systems. Several studies have reported *E*. *coli* fosfomycin resistance due to the defects in GlpT or UhpT [9,13,16]. In this study, mutations in the *glpT* gene were found in six *E*. *coli* strains, all of which resulted in amino acid substitutions in GlpT. In addition, single nucleotide deletion in the *glpT* gene, which would lead to a truncation of the GlpT sequence, was also detected.

Alteration in the chemical structure of fosfomycin by FosA_3_, a protein encoded by the *fosA*_*3*_ gene, was previously reported in three *E*. *coli* strains in Japan in 2010 for the first time [13]. In our study, over 80% of fosfomycin non-susceptible *E*. *coli* strains (69/86) harbored the *fosA*_*3*_ gene, which indicated that it is the main mechanism responsible for fosfomycin resistance in Mainland China. The *fosA*_*3*_ gene identified in 42% isolates (29/69) can transfer between different *E*.*coli* strains. Previous research has suggested that the *fosA*_*3*_ gene is encoded on a conjugated plasmid [21,22]. As the mobility of this gene may accelerate the dissemination of fosfomycin resistance around the world, future research is warranted to confirm this for *E*. *coli*.
